# Supplementary figures and images for: Whole exome sequencing reveals novel risk genes of pituitary neuroendocrine tumors
Source: PLoS One. 2022 Aug 26;17(8):e0265306. doi: 10.1371/journal.pone.0265306 (PMC9417189; doi:10.1371/journal.pone.0265306)

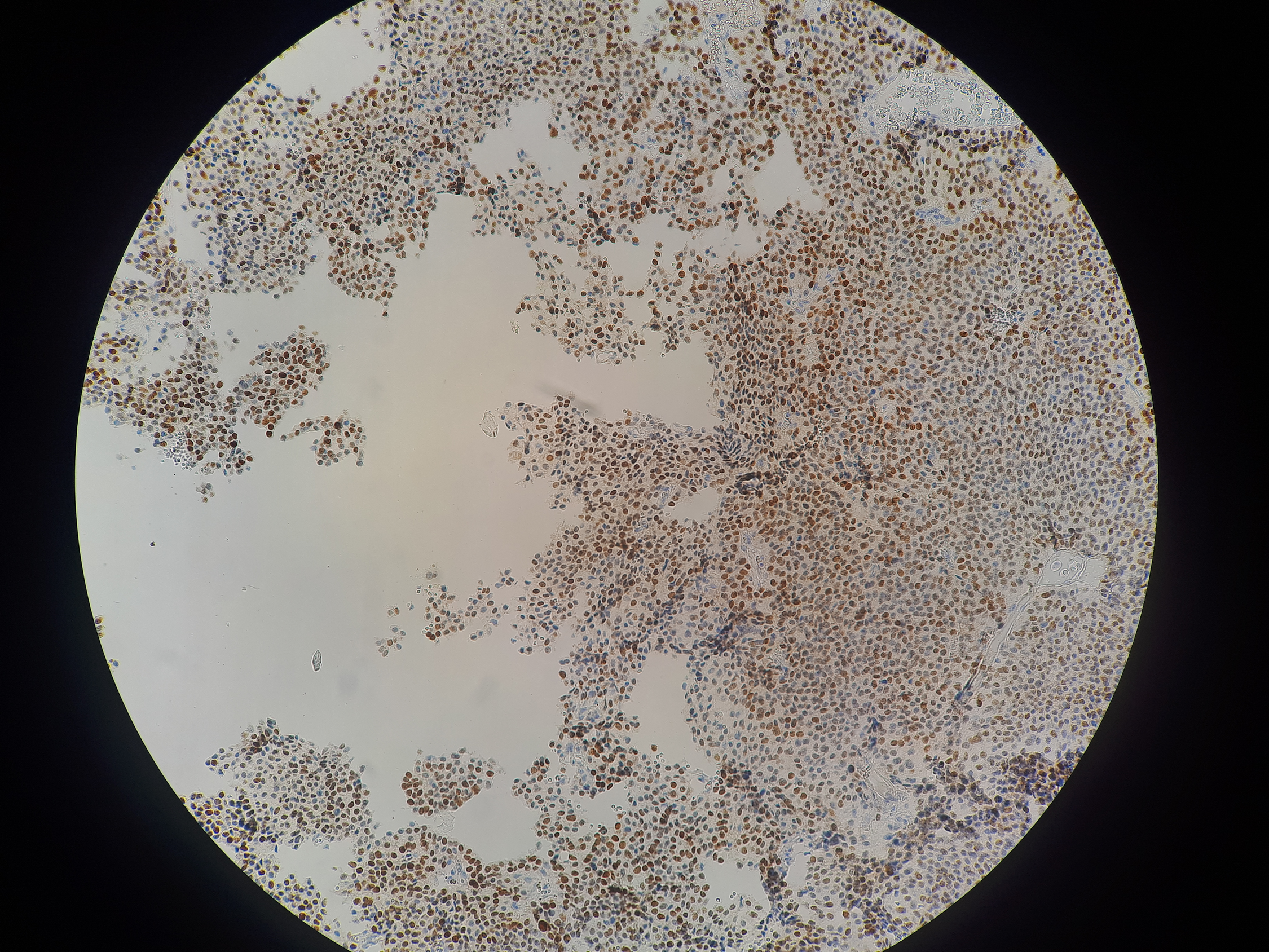

Supplement: S1 File — (ZIP) [file pone.0265306.s002.zip › PN02 TBX19.tif]

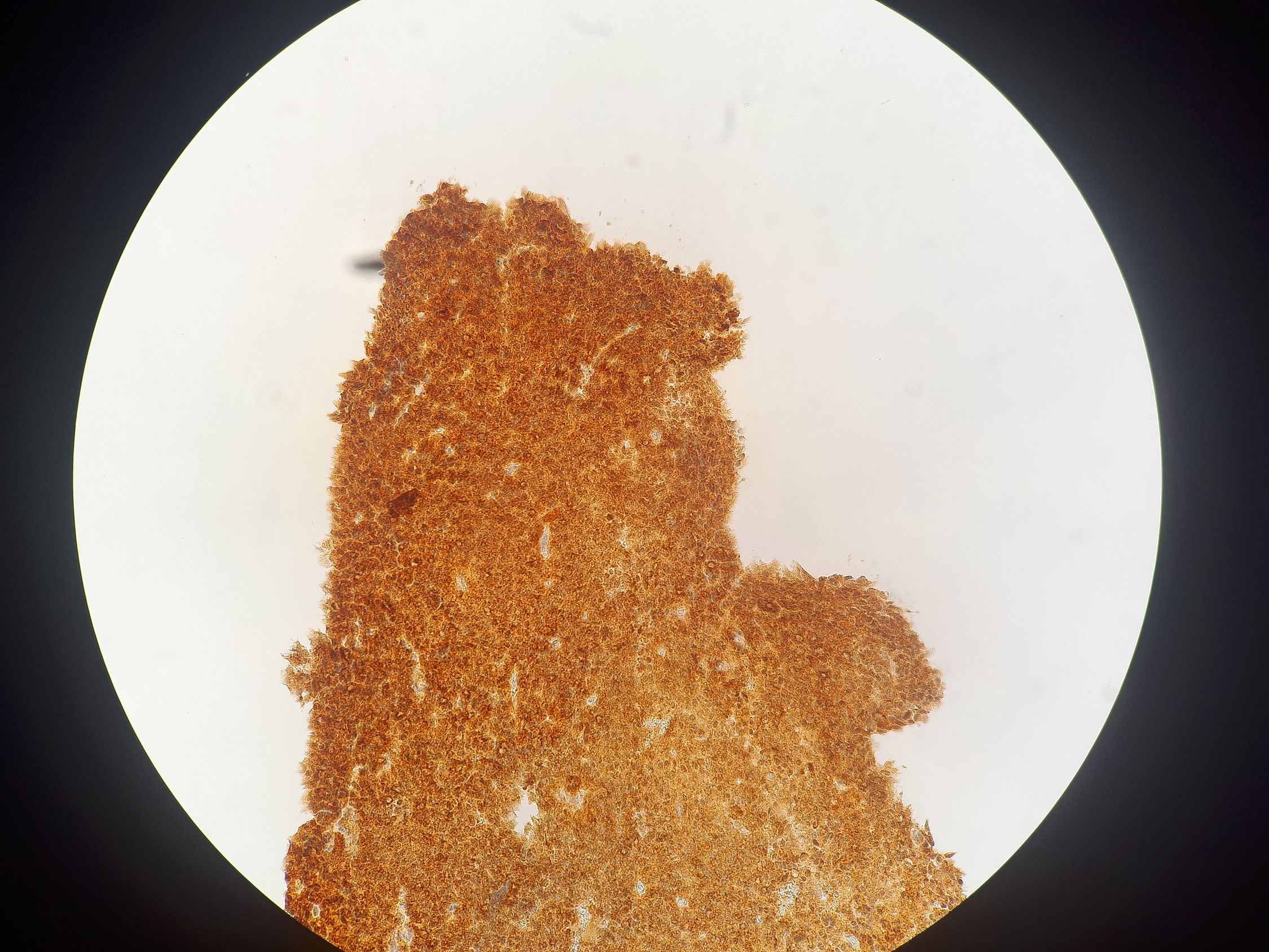

Supplement: S1 File — (ZIP) [file pone.0265306.s002.zip › PN10 NR5A1.tif]

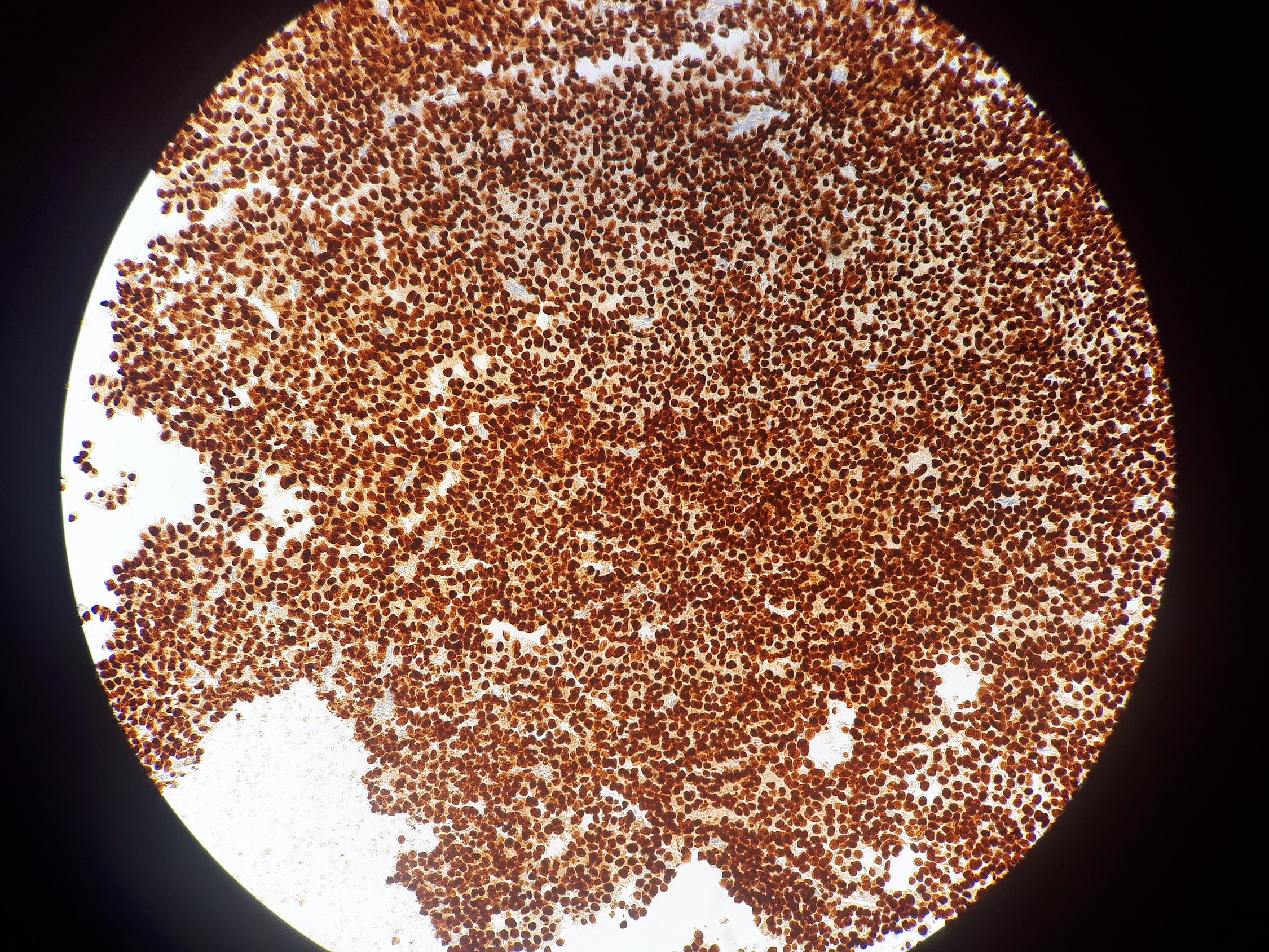

Supplement: S1 File — (ZIP) [file pone.0265306.s002.zip › PN14 PIT1.tif]
