# Supplementary material for: Whole exome sequencing reveals novel risk genes of pituitary neuroendocrine tumors
Source: PLoS One. 2022 Aug 26;17(8):e0265306. doi: 10.1371/journal.pone.0265306 (PMC9417189; doi:10.1371/journal.pone.0265306)
Supplement: Attachment — Submitted filename: Response to Reviewers_V2.doc [file pone.0265306.s003.doc]

**Response to Reviewers**

We thank editor and reviewers for considering this work and involvement to improve quality, relevance and readability of this research article.

**Journal requirements**

**1. Please ensure that your manuscript meets PLOS ONE's style requirements, including those for file naming.** The PLOS ONE style templates can be found at https://journals.plos.org/plosone/s/file?id=wjVg/PLOSOne_formatting_sample_main_body.pdf and https://journals.plos.org/plosone/s/file?id=ba62/PLOSOne_formatting_sample_title_authors_affiliations.pdf

We have used PLOS ONE style template for the main body of the text and formatted authors, affiliations and references according to journal requirements.

**2. Please provide additional details regarding participant consent. Specifically, in the ethics statement in the Methods and online submission information, please ensure that you have specified whether consent was informed.**

The consent was informed for all participants. We have corrected the ethics statement in the Methods:

“Two written informed consents have been obtained from the patients (1) broad consent for LGDB for use of biological material and medical data for human health and hereditary research, and (2) project-specific consent to the research of pituitary tumors, both studies have been approved by the Central Medical Ethics Committee of Latvia (protocol No. 22.03.07/A7 and 2/18-02-21, respectively).”

**3. We note that you are reporting an analysis of a microarray, next-generation sequencing, or deep sequencing data set. PLOS requires that authors comply with field-specific standards for preparation, recording, and deposition of data in repositories appropriate to their field. Please upload these data to a stable, public repository (such as ArrayExpress, Gene Expression Omnibus (GEO), DNA Data Bank of Japan (DDBJ), NCBI GenBank, NCBI Sequence Read Archive, or EMBL Nucleotide Sequence Database (ENA)). In your revised cover letter, please provide the relevant accession numbers that may be used to access these data. For a full list of recommended repositories, see http://journals.plos.org/plosone/s/data-availability#loc-omics or http://journals.plos.org/plosone/s/data-availability#loc-sequencing.**

We agree that it is important to deposit the sequencing data for ensuring reproducibility and transparent research. We have included the accession ID in the Methods section: “The sequencing data has been deposited to The European Genome-phenome Archive with access ID EGAC00001001730.”

**4. Please include captions for your Supporting Information files at the end of your manuscript, and update any in-text citations to match accordingly. Please see our Supporting Information guidelines for more information: http://journals.plos.org/plosone/s/supporting-information.**

We have added subheading: "Supporting Information captions" before list of references and text describing supporting information: "S1. Description of all somatic variants of PitNET exomes

S2. Example photographs of immunohistochemistry detection of PIT1 cell lineage marker, corticotroph cell lineage marker TBX19 (TPIT) and gonadotroph PitNET cell lineage marker NR5A1 (SF1)."

**Reviewers**

**1. Is the manuscript technically sound, and do the data support the conclusions?**

**Reviewer #1: Yes**

**Reviewer #2: Partly**

We have thoroughly revised the manuscript, reanalyzed data and in addition called variants using Strelka2 variant caller, while dropping results from Isaac-Starling (Illumina default) pipeline. Also description of the methods was revised to be cleared for readers.

**2. Has the statistical analysis been performed appropriately and rigorously?**

**Reviewer #1: Yes**

**Reviewer #2: No**

Upon remarks of the Reviewers we have considerably changed our approach in statistical analysis (see more detailed description below), and we hope that it makes our data interpretation more transparent and clear.

**3. Have the authors made all data underlying the findings in their manuscript fully available?**

**Reviewer #1: No**

**Reviewer #2: No**

We agree that it is important to deposit the sequencing data for ensuring reproducibility and transparent research. We have included the accession ID in the Methods section: “The sequencing data has been deposited to The European Genome-phenome Archive with access ID EGAC00001001730.”

**4. Is the manuscript presented in an intelligible fashion and written in standard English?**

**Reviewer #1: Yes**

**Reviewer #2: No**

Thank you for this comment we thoroughlychecked the manuscript for spelling and grammar mistakes. Upon futher request we are also open to send manuscript for the editing at professional scientific article editing service.

**Reviewer #1: In this manuscript, the authors described their finding of risk genes from patients with pituitary neuroendocrine tumors. With the whole exome-sequencing of 15 patients, the authors identified 284 somatic variants, some of which might be related to this disease. Comparing with previous publications, the authors conclude that two genes, NBPF1 and MUC12 are possible novel candidate genes for this disease. The written of this manuscript is precise and clear. This work is significant and will be helpful for future research and treatment of this disease. Thus, I suggest accepting this manuscript for publication if the authors can resolve my major concerns.**

We thank reviewer for careful consideration, evaluation and recognition of this work. Futher we include detailed description of the alterations suggested by the Reviewer.

**1. Two methods were utilized to call the somatic variants. However, the results are very different, one with 71 and one with 249 variants, and only 36 were shared by the two methods. How to explain this? I think the authors need to find the reason and change the settings to get more consistent results.**

Initially we were interested in SNV calling discrepancy. The first, Isaac-Starling method has been shown to be comparable in accuracy with much wider known GATK (Raczy, *et al*., 2013). We had suspicion that it might be biased towards reference because average fraction of alternative allele in heterozygotes was below 50%. Recent literature has confirmed this suspicion (https://doi.org/10.1093/bioinformatics/btaa514). This pipeline has been used in cancer publications previously (Burns, *et al*., 2018; Quigley, *et al*., 2018) and UK 100,000 Genomes Project (100KGP) (Turnbull, *et al*., 2018) but the settings are not directly comparable with GATK algorithm used in Dragen pipeline. O'Rawe, *et al*., 2013 and others have highlighted that concordance between various data analysis pipelines is low, but that does not mean that either is not correct. We originally wanted to show that different pipelines are supplementing each other, providing more information on somatic tumor variants in PitNETs. Due to these reasons we have refined our analysis considerably in this version of the manuscript.

In this revised version of the manuscript we have dropped Isaac-Starling method and replaced it with Strelka2 variant caller, showing high accuracy and reduced computing cost (Kim, *et al*. 2018, PMID 30013048). We are now reporting only variants where both variant callers (GATK and Strelka2) agree with the premise that problematic false positive variant calls included by the one of the algorithms will be excluded by the other.

It has been shown that alternate allele fraction in tumor could be low (< 20%) we did not consider appropriate to rely on Sanger sequencing for confirmation of the variants in tumor tissue We also think that fine tuning settings of variant calling software would make them susceptible for detecting the same false positive signals.

**2. For some mutations, especially those in NBF1 and MUC12, Sanger sequencing is necessary to verify the variants.**

We were sceptical of performing Sanger sequencing of variants with low alternate allele fraction, yet after reviewer’s comments we attempted to detect repeating variants with the Sanger sequencing for both variants in both samples.

The results with *MUC12* are ambiguous. One set of primers cannot detect mutation while other set of primers detected mutation in tumor sample (Fig 1 in this document).

Figure 1. *MUC12* chr7:100634821 Sanger sequencing results. Top PN01 forward primer, bottom, PN01 reverse primer. Position marked by red arrow.

On the other hand *NBF1* was even more surprising; instead of low fraction somatic call it was found that it is actually a germline variant.

This was one of the reasons we changed from reporting all variants of the both algorithms to only reporting somatic variants where GATK and Strelka2 showed the same result. The latest PitNET publications do not use Sanger sequencing to validate the somatic variants (Neou et al. 2020)

**3. One more thing, the raw data or vcf files are not public available. Not sure whether that’s required by PLOS One.**

The sequencing data has been deposited to The European Genome-phenome Archive with access ID EGAC00001001730.

**4. I suggest the authors list the allele frequencies (AFs) of variants in Table 1 and Table 2 on gnomAD. For example, AF of 1-16892193-A-T is 1% to 2% in the population, which is less likely a significant variant compared to others.**

We thank reviewer for careful consideration of this study. We had overlooked this issue ourselves and this prompted us to reanalyze the data and reconsider the results. The suggestion was taken into account and this information has been added to the tables.

**5. Line 209: change “The variant call file than was” to “The variant call file was then” or remove the word “than”.**

**Table 2: change “non coding” to “noncoding” or “non-coding”.**

**Line 308: change “NBPF1 contains somatic variant” to “NBPF1 contains somatic variants” or “NBPF1 contains a somatic variant”, depending on the number of variants.**

**Line 314, 333: change “PitNET sample from” to “PitNET samples from”.**

**Line 339: add “a” or “one” before “relatively hypermutated PitNET sample”.**

**Line 363: change “gnomAD frequence” to “gnomAD frequency”.**

**Line 385: change “was found in PN15” to “were found in PN15”.**

**Line 396: change “chromosomal abberations” to “chromosomal aberrations”.**

We have made the suggested corrections where they were lifted over from previous version of the manuscript.

**Reviewer #2: Peculis et al. performed whole-exome sequencing of 15 PitNET patients with normal/tumor pairs in attempt to identify the disease-causing mutations. The authors reported two "novel" mutated genes, MUC12 and NBPF1, while both were mutated in only two patients and at low allelic frequency (likely subclonal). Given the low prevalence amongst patients and mutated alleles within tumors and also the lack of functional validation, I don't think they are the drivers of PitNET development, although they might play some role in tumor evolution after tumor transformation.**

We thank reviewer for careful evaluation of this work. We performed mutation calling using different methods and saw that such approach is increasing the available amount of somatic variation, that are called automatically, therefore increasing precision. We agree that discovered somatic variants in *MUC12* and *NBPF1* are unlikely to cause tumor development and as Sanger sequencing has revealed since, are indeed questionable somatic events. The emphasis was that we think these genes show recurrent somatic event, therefore, are mentionable.

Due to new analysis of the data and change in reporting concept (due to issues raised by reviewers), we have omitted *MUC12* and *NBPF1* variants from current version of the manuscript.

**1. Technically, this SNV detection and analysis in this paper is very pool [poor] while just following the Illumina's in-house pipeline, which is not the standard of cancer genomic analysis.**

Due to reviewers concerns and additional literature review we switched from Illumina in-house pipeline Isaac-Starling to Strelka2 variant caller, which shows high accuracy and reduced computing cost (Kim, *et al*. 2018, PMID: 30013048)

Literature review for Isaac-Starling method revealed that this method is comparable in accuracy with much wider used GATK (Raczy, *et al*., 2013),but it is biased towards reference because average fraction of alternative allele in heterozygotes was below 50%. (https://doi.org/10.1093/bioinformatics/btaa514). Still this pipeline has been used in cancer publications previously (Burns, *et al*., 2018; Quigley, *et al*., 2018) and UK 100,000 Genomes Project (100KGP) (Turnbull, *et al*., 2018).

We revised Methods section to note that Dragen is hardware accelerated wrapper of GATK which has been praised by developers of GATK themselves (https://gatk.broadinstitute.org/hc/en-us/articles/360039984151-DRAGEN-GATK-Update-Let-s-get-more-specific).

**2. What's more, CNA analysis is not even performed.**

We performed search of CNA (CNV) using CoNIFER and XHMM algorithms and had negative results. Confirming that CNV call from exome sequencing data is problematic (M. Fromer and S. M. Purcell, 2015). As we didn't have array genotyping data for these samples to benchmark CNVs and support this negative finding, they were initially not included in the manuscript. Also at the first analysis we used Illumina in-house pipeline’s structural variation detection with MANTA algorithm, but due to low concordance between normal and tumor samples the results were dropped from the manuscript. To clarify we added following text in the "Data analysis" section: "Copy number analysis using CoNIFER and XHMM algorithms did not reveal presence of CNA in analysed samples."
